# Supplementary material for: Hippocampal OLM interneurons regulate CA1 place cell plasticity and remapping
Source: Nat Commun. 2025 Nov 11;16:9912. doi: 10.1038/s41467-025-64859-0 (PMC12606138; doi:10.1038/s41467-025-64859-0)
Supplement: Supplementary file 1 — Supplementary information [file 41467_2025_64859_MOESM1_ESM.pdf]

**Supplementary Information for Udakis et al.**

**Supplementary Figures**

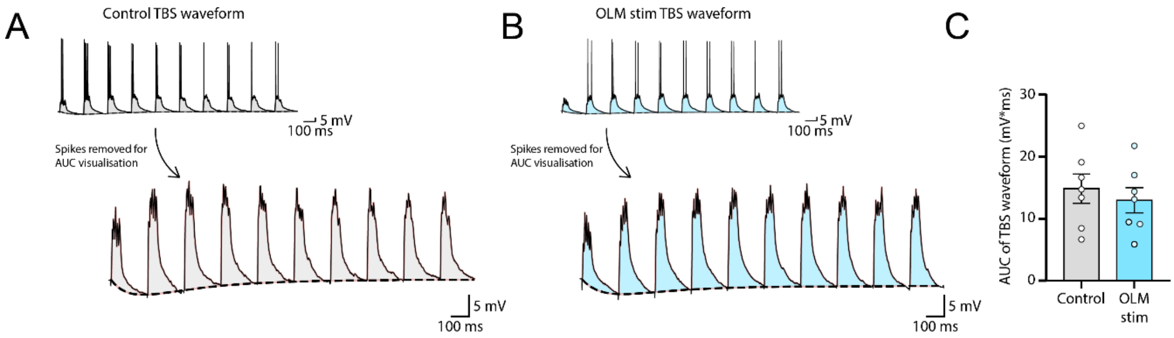


**Figure S1 – OLM activation during LTP induction by theta burst stimulation (TBS) does not alter the degree of somatic depolarization.**

Example traces of somatic membrane potential during TBS in control (A) and with OLM stimulation (B). Bottom traces show responses with action potentials deleted and the AUC indicated in shaded region.

C). Quantification of AUC for all LTP experiments in Figure 1 shows no significant difference between control and OLM stimulation conditions. Data presented as mean ± sem.

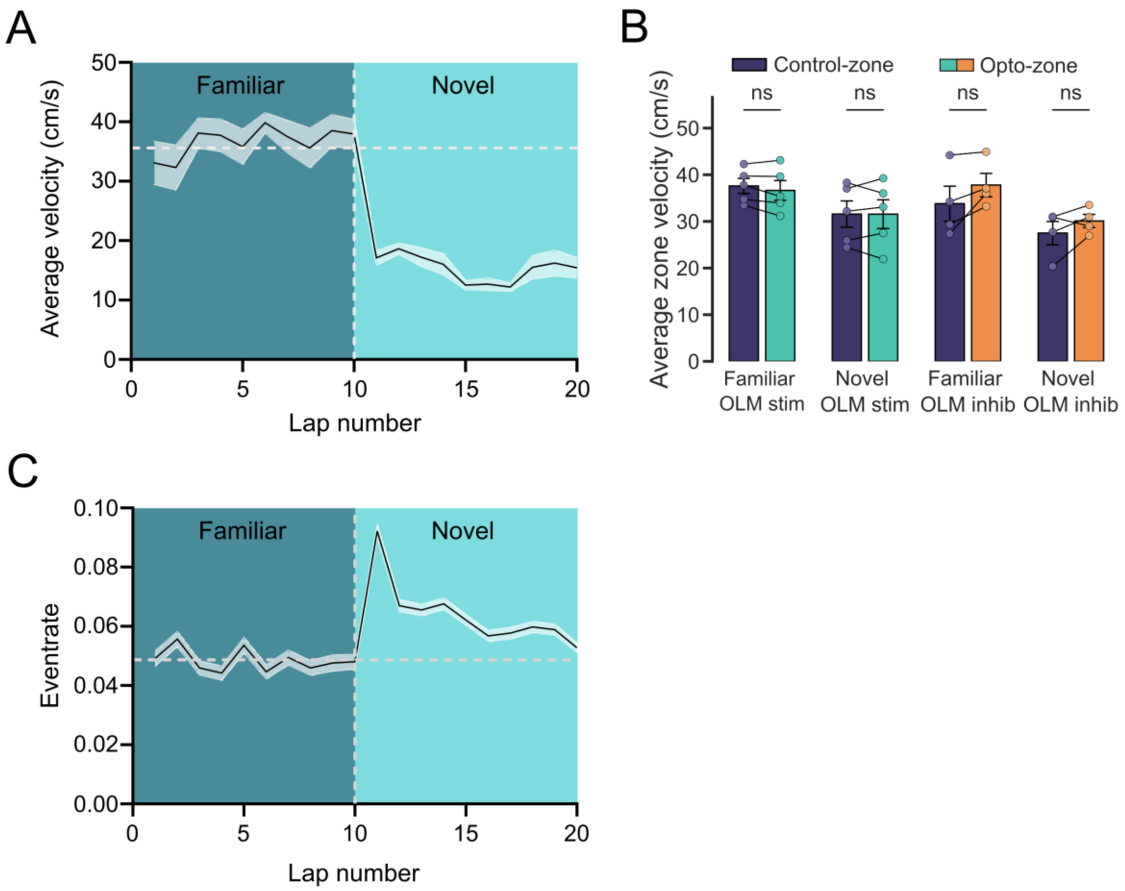


**Figure S2 – Animal velocity during exploration of familiar and novel environments**

A) Lap by lap velocity of mice as they traverse a familiar environment (last 10 laps) and a novel environment (first 10 laps) averaged across animals (n = 13 mice).

B) Average mouse velocity within the control and Opto stimulation zones for the corresponding experiments in Figures 4 and 5. OLM stim n = 5 mice, OLM inhib. n = 4 mice.

C) Lap by lap pyramidal cell event rates as mice traverse a familiar environment (last 10 laps) and a novel environment (first 10 laps), n = 5195 cells (from 9 mice).

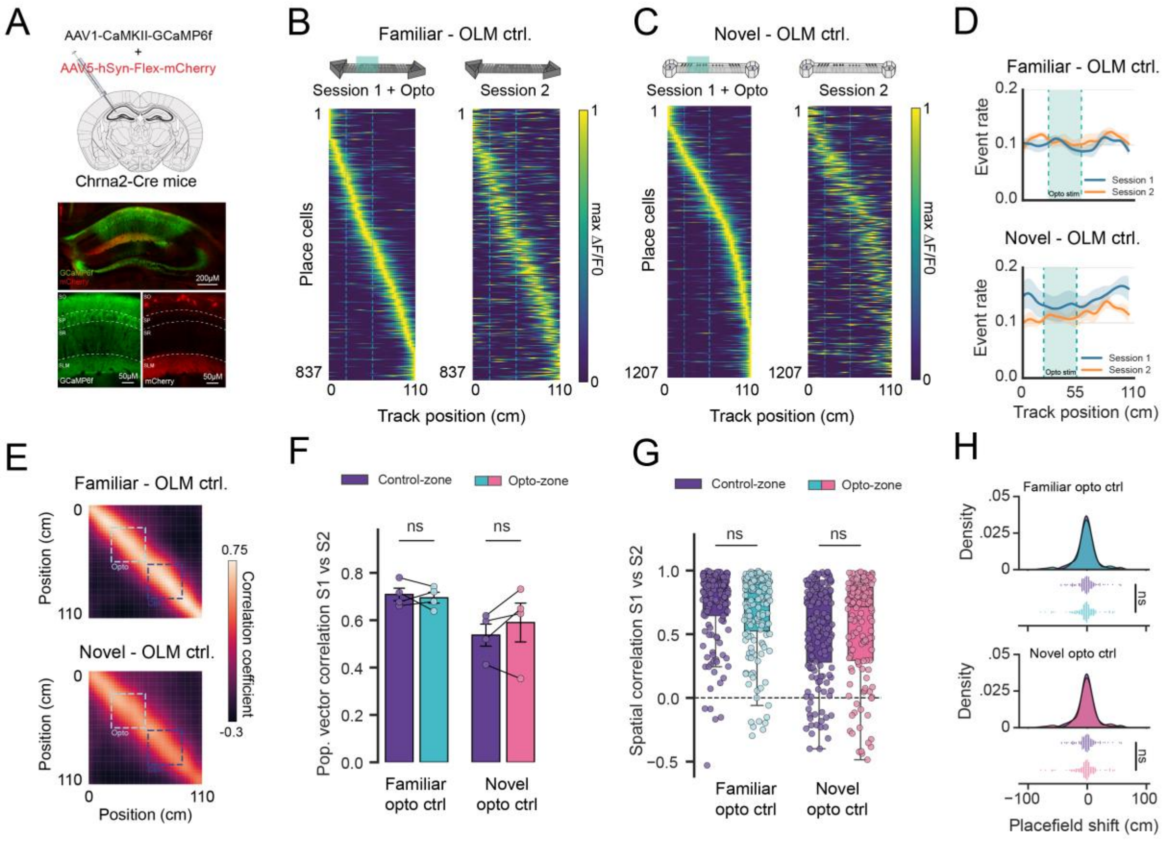


**Figure S3 – mCherry control for optogenetic stimulation of OLM interneurons in familiar and novel environments shown in Figures 4 and 5.**

(A) Chrna2-cre mice were injected into the dorsal hippocampus with viral constructs to express GCaMP6f in pyramidal and mCherry into OLM interneurons. Example expression of mCherry in OLM interneurons.

(B) Place cells acquired in a familiar environment (session 1) with light stimulation on opto zone. Activity of the same cells in session 1 during the second exposure to the familiar environment (session 2).

(C) Same as B except for the first exposure to a novel environment.

(D) $Ca^{2+}$ event rate of all recorded neurons along the length of the track during the exploration of familiar or novel environments (Familiar n = 4 mice, Novel n = 4 mice)

(E) Population vector correlation matrix between activity rate maps in session 1 vs session 2 for familiar and novel environments in B,C, averaged across animals.

(F) Average population vector correlation taken as average diagonal correlation for each track zone, (Control-zone vs Opto-zone). (Familiar n = 4 mice, Novel n = 4 mice).

(G) Spatial correlation values between place cells in session 1 and the activity of the same cells in session 2, for both control and optogenetic zones, in familiar and novel environments.

(H) Place field location shifts from place cells in session 1 vs place field location of same cells in session 2.

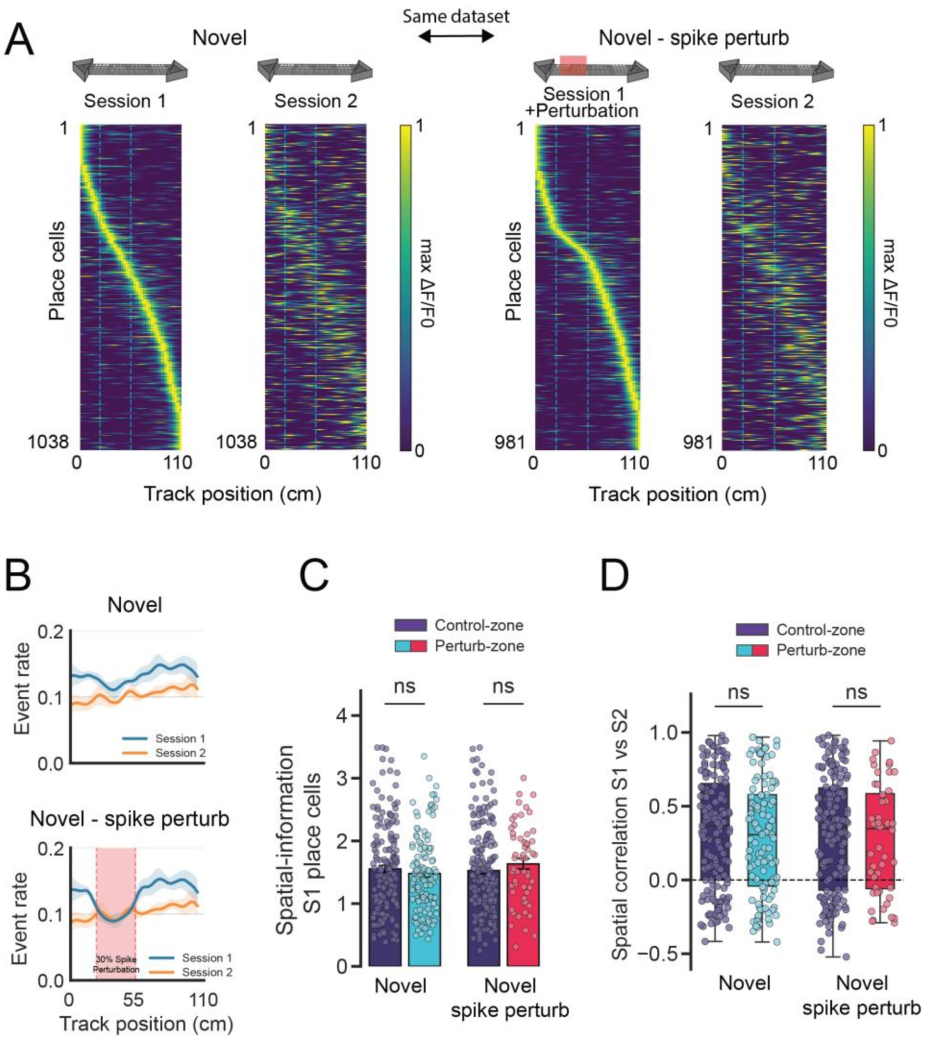


**Figure S4 – Posthoc spike perturbation simulation in novel environment control for data shown in Figure 4.**

(A) Left, a control mCherry dataset in the absence of light stimulation, place cells activity data are shown for a novel environment (session 1) and the activity of the same cells during the second exposure to the same environment (session 2). Right, in the OLM stimulation zone (perturbation zone), spikes were posthoc removed randomly at 30% probability from the same dataset prior to place cell analysis to mimic the effect of OLM interneuron stimulation.

(B) Event rates along the length of the track for both the novel and the novel + perturbation datasets (n = 4 mice).

(C) Left, spatial information values between place cells in session 1 and the activity of the same cells in session 2, for both control and perturbation zones, in novel and the novel + perturbation datasets.

(D) Same as C except for spatial correlation values.

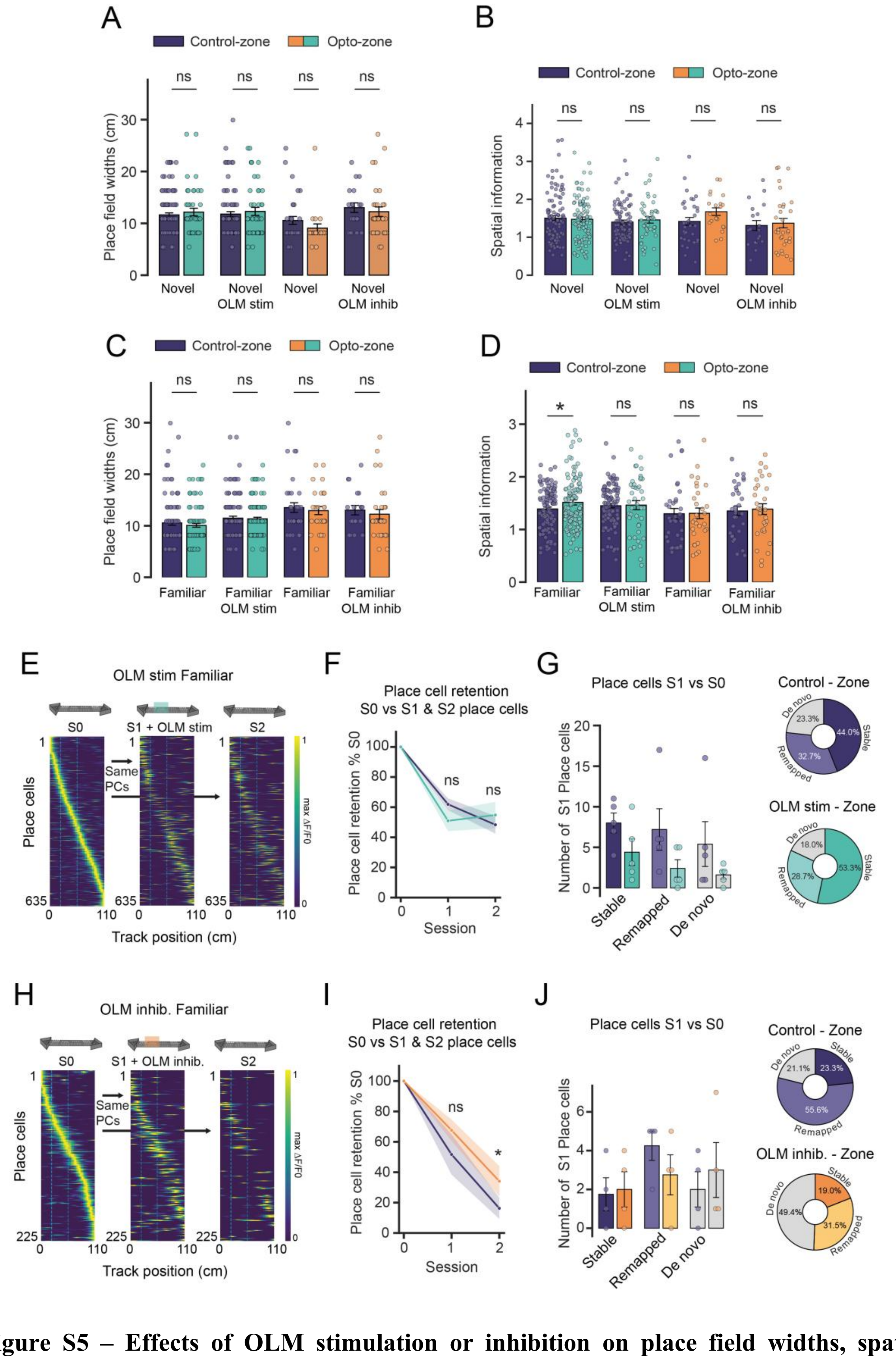


**Figure S5 – Effects of OLM stimulation or inhibition on place field widths, spatial information and place cell retention.**

Place field widths (A,C) and spatial information (B,D) in control or opto stim zones for OLM stimulation or inhibition in novel (A,B) or familiar (C,D) environments. Supplementary information related to Figures 4 and 5.

(E) Place cell rate map retention between three explorations of a familiar track (sessions 0-2) with OLM activity stimulation in middle session (session 1) related to Figure 5. Place cell rate maps from place cells in session 0 aligned to activity of the same cells that are also place cells in session 1 or session 2.

(F) Quantification of E, number of place cells in session 1 and session 2 retained from session 0 for each animal. (Paired t-test, n = 5 mice). Data presented as mean values ± S.E.M.

(G) Place cell retention for place cells in session 1 vs same cells in session 0. Stable place cells: place cells in session 1 also a place cell in session 0 encoding the same location. Remapped place cells: place cells in session 1 also a place cell in session 0 but encoding a different location. *De novo* place cells: place cell in session 1 that was not a place cell in session 0. Average percentage of, stable, remapped and *de novo* place cells in optogenetic stimulation and control zones shown on right. Data presented as mean values ± S.E.M.

(H-J) Same as E-G but with OLM interneuron inhibition in the opto zone. Related to Figure 5.

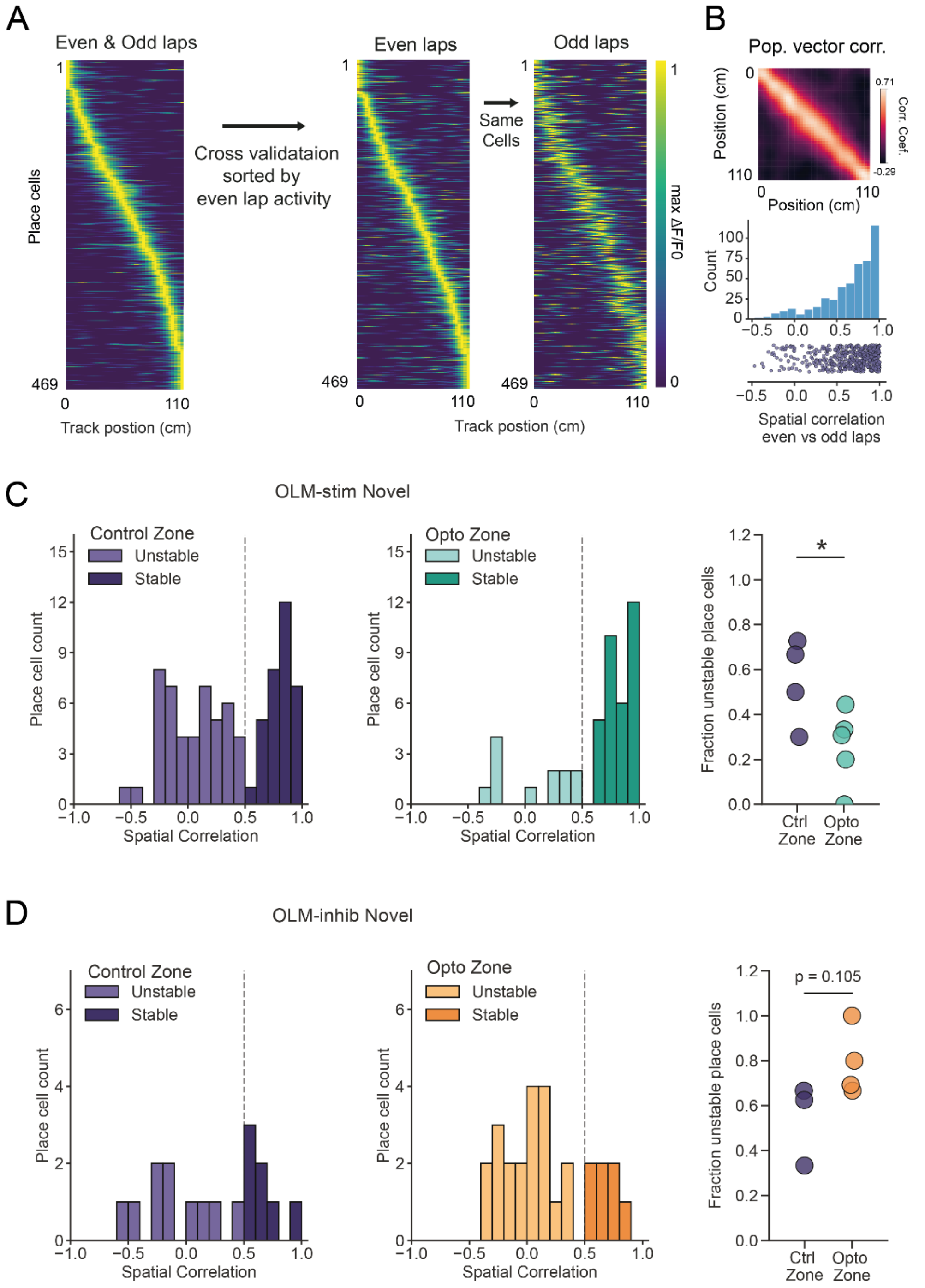


**Figure S6 – Cross-validation of place fields related to Figures 4,5&6 and proportions of stable and unstable place cells after OLM stimulation or inhibition in a novel environment related to Figure 4.**

(A) Average place cell population (left) split into even laps (middle) and odd maps sorted same as even laps (right) for mice in a familiar environment.

(B) Population vector correlations (top) and spatial correlations (bottom) between even and odd laps.

(C) Spatial correlation distribution for place cells in control and opto zones compared between sessions 1 and 2 for OLM stimulation in a novel environment shown in Figure 4. Classification of stable or unstable cells was set using a threshold of 0.5 spatial correlation. Proportion of unstable place cells for each mouse is plotted on the right.

(D) Same as C but for OLM inhibition.
